# Effects of Intraoperative Ventilation Strategies on Ventilation Inhomogeneity and Inflammatory Response in Pediatric Cardiac Surgery—A Randomized Pilot Study

**DOI:** 10.1111/pan.70066

**Published:** 2025-10-23

**Authors:** Charlotte Billstein, Alina Schenk, Mathieu Vergnat, Patrick Jakobs, Stilla Frede, Christian P. Putensen, Thomas Muders, Ehrenfried Schindler

**Affiliations:** ^1^ Department of Anesthesiology and Intensive Care Medicine University Hospital Bonn Bonn Germany; ^2^ Institute of Medical Biometry, Informatics and Epidemiology University Hospital Bonn Bonn Germany; ^3^ Department of Pediatric Cardiac Surgery University Hospital Bonn Bonn Germany

**Keywords:** cardiopulmonary bypass, electrical impedance tomography, inflammatory response, lung‐protective ventilation, pediatric cardiac surgery, ventilation inhomogeneity

## Abstract

**Background:**

Respiratory arrest during cardiopulmonary bypass (CPB) in pediatric cardiac surgery risks lung dysfunction including derecruitment, atelectasis, and inflammation. Continuous positive airway pressure (CPAP) and lung‐protective ventilation (LPV) during aortic cross‐clamping show inconsistent results in mitigating these risks.

**Aims:**

To investigate whether LPV during aortic cross‐clamping under CPB affects postoperative respiratory mechanics and ventilation inhomogeneity compared to apnea or CPAP.

**Methods:**

This prospective, randomized pilot study compared three ventilation strategies during aortic cross‐clamping under CPB: apnea, CPAP (5 mbar), and LPV. LPV was standardized using pressure‐controlled ventilation at a positive end‐expiratory pressure of 5 mbar, individualized driving pressure (20% of the pre‐cross clamp inspiratory pressure), and age‐adjusted respiratory rate. Recruitment maneuvers were applied at the end of CPB. Respiratory mechanics were assessed. Ventilation distribution was measured preoperatively and postoperatively under spontaneous breathing and mechanical ventilation using Electrical Impedance Tomography. Blood was analyzed pre‐ and postoperatively for pulmonary and systemic inflammatory markers. Feasibility of LPV was assessed. Statistical analysis used linear mixed‐effects models.

**Results:**

Driving pressure increased (11.8 (2.6) to 12.9 (2.6) mbar) and dynamic compliance decreased (9.9 (7.3) to 8.5 (7.4) Pa L^−1^) statistically significantly preoperatively to postoperatively. The number of ventilated pixels increased statistically significantly from spontaneous breathing (408.2 (77.2)) to mechanical ventilation (495.1 (44.9)) and returned toward baseline postoperatively (433.9 (72.6)). The Center of Ventilation shifted statistically significantly ventrally during mechanical ventilation (0.491 (0.039) to 0.442 (0.027)) and normalized afterward (0.485 (0.037)). These changes were unaffected by the ventilation strategy. Biomarker analysis showed no statistically significant changes between groups. LPV during aortic cross‐clamping was feasible.

**Conclusion:**

In this pilot study, ventilation strategies did not differ in their effect on ventilation distribution, respiratory mechanics, or inflammatory markers when recruitment maneuvers were uniformly applied after CPB. LPV was feasible.

**Trial Registration:**

German Clinical Trials Register: DRKS00030219; https://drks.de/search/de/trial/DRKS00030219

## 1 Introduction

Congenital heart disease (CHD) often requires cardiac surgery involving cardiopulmonary bypass (CPB). As CPB guarantees blood oxygenation and carbon dioxide elimination [1], lung ventilation is typically interrupted during aortic cross‐clamping. Respiratory arrest promotes lung injury and remodeling due to lung derecruitment and atelectasis formation [2], which can lead to short‐ and long‐term pulmonary dysfunction by increasing pulmonary and systemic inflammatory responses [3, 4]. Pediatric patients face additional risks due to immature alveolar structure and ongoing lung development [3], generating particular interest in improving ventilation strategies and minimizing pulmonary injury. Established ventilation strategies during aortic cross‐clamping include continuous positive airway pressure (CPAP) [5] and lung‐protective ventilation (LPV) [6], followed by recruitment maneuvers to reopen poorly ventilated areas [7]. While these ventilation strategies have shown positive effects on postoperative lung function [8], other studies suggest minimal significant differences and no long‐term benefits [9], highlighting the need for comprehensive investigation.

To the best of our knowledge, this is the first study evaluating the effects of different intraoperative ventilatory strategies during aortic cross‐clamping on ventilation inhomogeneity—assessed via Electrical Impedance Tomography (EIT)—and epithelial injury, as indicated by systemic and pulmonary inflammatory markers in pediatric cardiac surgery, while investigating whether LPV interferes with cardiac surgical procedures.

We hypothesize that LPV during CPB reduces postoperative ventilation inhomogeneity and systemic and pulmonary inflammation by preventing atelectasis formation and its associated inflammatory response. Primary outcomes include pre‐ and postoperative ventilation inhomogeneity following intraoperative apnea at ambient pressure, CPAP, and LPV using standard EIT parameters (number of ventilated pixels, Center of Ventilation, Global Inhomogeneity Index), as well as respiratory mechanics with a focus on driving pressure and compliance. Secondary outcomes comprise the feasibility of LPV during aortic clamping, pulmonary epithelial injury assessed via specific biomarkers (receptor for advanced glycation end‐products (RAGE) and surfactant protein D (SP‐D)), and markers of systemic inflammation.

## 2 Methods

### 2.1 Study Design

This prospective, randomized, blinded monocentric pilot study was conducted at the German Pediatric Heart Center of the University of Bonn, in collaboration with the Department of Pediatric Anesthesiology at University Hospital Bonn. The study adhered to the Declaration of Helsinki guidelines and received approval from the Ethics Committee of the Medical Faculty, Rheinische Friedrich‐Wilhelms‐University of Bonn (Ethics Approval No: 251/22, July 2022) and was registered in the German Clinical Trials Register (DRKS00030219).

### 2.2 Inclusion and Exclusion Criteria

To be included in the study, patients had to undergo elective cardiac surgery with CPB and aortic cross‐clamping. Exclusion criteria were body weight outside the range of 4–20 kg, physical status according to the American Society of Anesthesiologists (ASA) > 4, and contraindications to EIT, such as pacemaker implantation.

### 2.3 Anesthesia

On the day of surgery, all patients older than 6 months routinely received premedication with midazolam (0.5 mg kg^−1^). The first measurement via EIT during spontaneous breathing was performed under light sedation with a bolus of propofol (1–2 mg kg^−1^). Anesthesia was then induced with a bolus of 2–4 mg kg^−1^ propofol (omitted in children under 6 months), 50–100 μg fentanyl, and 0.15–0.20 mg kg^−1^ cisatracurium. Nasal intubation was performed in patients younger than 2 years, whereas oral intubation was used in patients aged 2 years and above. Intraoperative anesthesia was maintained with remifentanil (20–40 μg kg^−1^ h^−1^) and sevoflurane (end‐expiratory concentration 1.5–2.0 vol%) with flow of fresh gas < 2.0 L min^−1^ and fraction of oxygen in fresh gas ranging from 0.25 to 0.45. Emergence from anesthesia was performed following a standardized protocol: discontinuation of sevoflurane and remifentanil, transitioning analgosedation from remifentanil to piritramide (an opioid analgesic similar to morphine, dosed at 0.2–0.3 mg kg^−1^) with additional metamizole (a non‐opioid analgesic and antipyretic, dosed at 15 mg kg^−1^), continuous sedation maintained with dexmedetomidine (0.5–1.0 μg kg^−1^ h^−1^).

### 2.4 Ventilation Strategies

Following tracheal intubation, pressure‐controlled mechanical ventilation was performed using a positive end‐expiratory pressure (PEEP) of 5 mbar and an individualized peak inspiratory pressure (PIP) to result in a tidal volume (*V*_*T*_) of 6–8 mL kg^−1^, while the rate of respiration was age‐adjusted and CO_2_‐controlled. Inspired fraction of oxygen (FiO_2_) was maintained as low as clinically feasible, between 0.25 and 0.45.

Patients were then randomized into three groups with different ventilation strategies during aortic cross‐clamping in total CPB. Group 1 received no ventilation at ambient airway pressure (apnea). Group 2 received apnea at a CPAP level of 5 mbar. Group 3 received LPV, with pressure‐controlled mechanical ventilation, using PEEP of 5 mbar. Driving pressure for LPV was individualized as 20% of the initial PIP (set prior to aortic cross‐clamping). The respiratory rate remained at the age‐adjusted value established before cross‐clamping. Feasibility of LPV was defined as the ability to maintain target ventilation parameters without interfering with surgical procedures. If LPV interfered with surgical procedures by disturbing the surgeon through lung‐dependent movements, PIP was reduced accordingly; if interference persisted, LPV was discontinued entirely. Recruitment maneuvers were manually performed at the end of CPB, using 10 insufflations up to 25–30 mmHg with a ventilation bag.

After surgery and chest closure, settings for ventilation matched preoperative settings. Following the restoration of spontaneous breathing, patients were extubated and received oxygen (2 L min^−1^) via nasal cannula.

### 2.5 Measurements

#### 2.5.1 Global Respiratory Mechanics

PEEP, PIP, and dynamic compliance (Cdyn) were continuously obtained from the integrated anesthesia machine monitor. Driving pressure was calculated as the difference between PIP and PEEP. Mechanical power (MP) was determined according to the method described by Becher et al. [10].

#### 2.5.2 EIT Data Acquisition

Measurements using EIT were performed in the supine position to analyze ventilation distribution using the PulmoVista 500 EIT device (Draeger Medical Deutschland GmbH, Lübeck, Germany) as previously reported [11]. A 16‐electrode pediatric silicone belt (size 4XS to XS) was placed between the 3rd and 5th intercostal spaces, with belt position marked. Measurements using EIT were conducted in adjacent drive configuration at 80–130 kHz and a frame rate of 50 Hz. Global respiratory mechanics were measured and analyzed by the ventilator unit of the anesthesia machine and automatically transferred to the EIT system. At each point of measurement, a stable 4‐min phase of spontaneous breathing or controlled mechanical ventilation was recorded for offline analysis.

#### 2.5.3 EIT Data Generation

Following image reconstruction applying a modified Newton–Raphson algorithm [12], global and regional impedance/time curves were analyzed using a custom‐made software (MATLAB 21a; The MathWorks Inc.) as previously reported [11]. After low‐pass filtering (*f*_*c*_ = 60 Hz) and masking (10% threshold), 10 consecutive breaths were averaged from each phase of measurement to calculate mean functional images of the tidal variation (TV) [13].

TV images were divided into four horizontal regions (ventral, mid‐ventral, mid‐dorsal, and dorsal). The total number of ventilated pixels, serving as a surrogate parameter for the ventilated lung area [14], was reported as an absolute value for all regions. The Center of Ventilation (CoV) was calculated from TV images to assess the horizontal (CoVx) and vertical (CoVy) distribution of ventilation [15].

Global Inhomogeneity (GI) Index, an established measure of ventilation distribution across the lungs, was calculated as described by Zhao and others [16].

#### 2.5.4 Collection of Blood Samples and Analysis Using Multiplex Assay

The effects of ventilation strategies on pulmonary and systemic inflammation were examined by analyzing blood plasma (sampled from a catheter placed in the internal jugular central vein) using the R&D Systems Luminex multiplex immunoassay (Bio‐Techne). This immunoassay enables measurement and analysis of immune complex formation through antigen–antibody reactions [17]. Luminex immunoassay data were analyzed using the FLEXMAP 3D system, generating CSV files imported into Quantist Luminex Data Analysis Software Version 1.0.1 (Bio‐Techne).

To evaluate lung injury and inflammation, we examined interleukin‐6 (IL‐6), angiopoietins, RAGE, and SP‐D. Angiopoietins (Ang), particularly the Ang‐2/Ang‐1 ratio, are linked to inflammation and capillary leak syndrome [18], while RAGE and SP‐D are indicators of injury and damage to the lungs [19, 20]. Detailed reasons for selecting these parameters are provided in the Supporting Information (p. 1–2).

#### 2.5.5 Blood Gas Analysis

Arterial blood gas values were measured using standard technology.

### 2.6 Protocol

**FIGURE 1:**
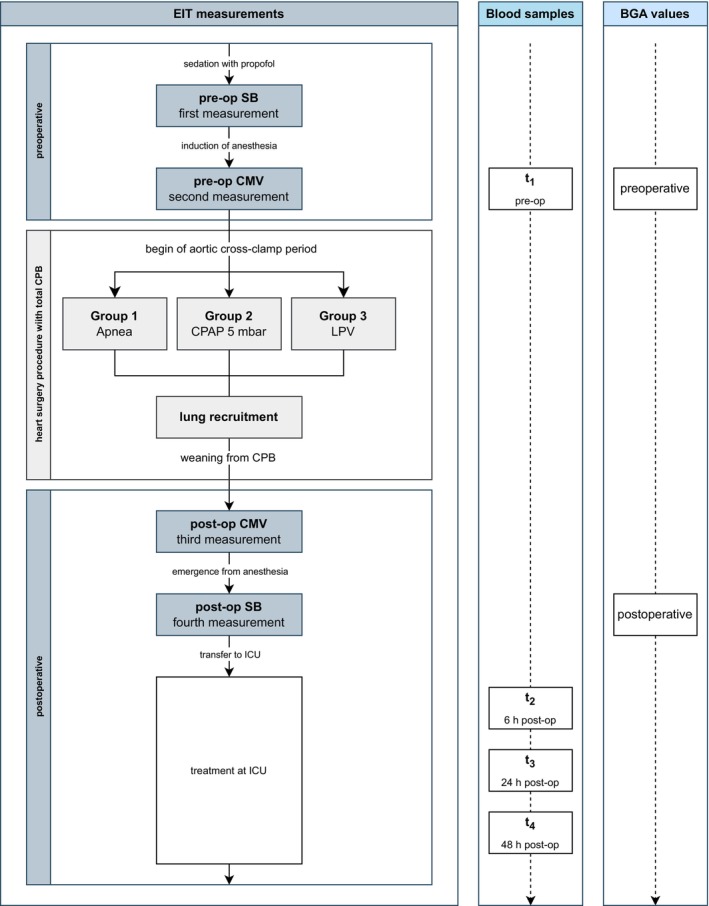
Study design. BGA, blood gas analysis; CMV, controlled mechanical ventilation; CPAP 5 mbar, continuous positive airway pressure at 5 mbar; CPB, cardiopulmonary bypass; EIT, Electrical Impedance Tomography; ICU, intensive care unit; LPV, lung‐protective ventilation; post‐op, postoperative; pre‐op, preoperative; SB, spontaneous breathing; t_1_, preoperative time point; t_2_, 6 h postoperative; t_3_, 24 h postoperative; t_4_, 48 h postoperative.

All patients underwent a standardized measurement protocol comprising preoperative, intraoperative, and postoperative phases (Figure 1). Four measurements using EIT were conducted. Preoperatively, the first measurement (pre‐op SB) was performed under propofol sedation during spontaneous breathing, and the second measurement (pre‐op CMV) followed induction of anesthesia under pressure‐controlled mechanical ventilation. Postoperatively, the third measurement (post‐op CMV) was taken under pressure‐controlled mechanical ventilation, and the fourth measurement (post‐op SB) was taken after emergence from anesthesia and return to spontaneous breathing. No measurements using EIT were performed during the period of aortic cross‐clamping with randomized intraoperative ventilation strategies. Arterial blood gas values were recorded with the second and the fourth measurements using EIT. Blood samples to measure inflammatory effects were collected at four time points: preoperatively (t_1_), and postoperatively at 6 h (t_2_), 24 h (t_3_), and 48 h (t_4_) after chest closure.

### 2.7 Randomization and Enrollment of Patients

Patients were randomized using a pre‐generated computer list. Each patient was consecutively assigned a number corresponding to one of three intervention groups based on this sequence. Enrollment occurred after confirming inclusion criteria, providing study information, and obtaining written informed consent from legal guardians. Both patients and guardians were blinded to the assigned intervention. Recruitment of patients occurred from September 2022 to July 2023.

### 2.8 Statistical Analysis

The sample size for this pilot study was determined based on feasibility, with 20 participants per group selected.

Statistical analysis was performed using the R language and environment for statistical computing (version 4.3.2). Descriptive statistics for continuous variables are presented as mean and standard deviation (Mean (SD)) and median with interquartile range (Median [IQR]). Categorical variables are presented as absolute numbers and valid frequencies.

Global respiratory mechanics, ventilation, inhomogeneity parameters, and arterial blood gas values were analyzed using linear mixed‐effects models, accounting for age, intervention groups, measurement time points, and their interaction. EIT data were similarly analyzed, considering age, regions of interest (ROIs), intervention groups, measurement time points, and their interactions. Venous blood samples were analyzed with baseline adjustment using preoperative values and their interaction with measurement time points as covariates. The influence of covariates and interactions in the linear mixed‐effects models was globally tested using F‐tests. The significance level for all tests was set at 5%. Given the exploratory nature of the study, no adjustments were made for multiple testing.

## 3 Results

### 3.1 Study Population

Epidemiological data of the participating children are presented in Table 1, with additional details provided in Table S1. The mean age of the cohort was 20.1 (19.1) months, with children in the LPV group being older (24.1 (16.9) months) compared to the apnea group (18.9 (23.1) months) and the CPAP 5 group (17.4 (17.4) months). The mean weight was 9.0 (3.8) kg. The cohort comprised an equal distribution of male and female patients. All participants had an ASA physical status of 3 or 4, with 36 classified as ASA 3 and 24 as ASA 4. Ventricular septal defect repair was the most frequently performed procedure in all groups (*n* = 17), followed by repair of Tetralogy of Fallot (*n* = 11), atrioventricular septal defect (*n* = 10; CPAP 5: 5, LPV: 3, apnea: 2), and atrial septal defect (*n* = 8; LPV: 4, apnea: 2, CPAP 5: 2).

**TABLE 1:** Epidemiological data of the participating children categorized into three intervention groups.

Parameter	Measure	Apnea (*n* = 19)	CPAP 5 (*n* = 21)	LPV (*n* = 20)	Total (*n* = 60)
Age (months)	Mean (SD)	18.9 (23.1)	17.4 (17.4)	24.1 (16.9)	20.1 (19.1)
	Median [IQR]	7.1 [5.6, 23.2]	7.2 [6.1, 24.5]	24.0 [6.1, 35.8]	8.0 [6.0, 32.2]
Weight (kg)	Mean (SD)	8.7 (4.4)	8.6 (3.5)	9.8 (3.4)	9.0 (3.8)
	Median [IQR]	6.9 [5.6, 11.4]	7.2 [6.5, 10.1]	9.7 [6.9, 12.3]	7.6 [6.0, 12.1]
Height (cm)	Mean (SD)	73.6 (18.1)	74.2 (13.2)	80.3 (14.7)	76.0 (15.4)
	Median [IQR]	67.0 [61.5, 81.5]	67.0 [65.0, 80.0]	80.5 [67.0, 89.0]	69.0 [64.8, 87.0]
Sex					
M	*n* (%)	10 (53%)	11 (52%)	9 (45%)	30 (50%)
F	*n* (%)	9 (47%)	10 (48%)	11 (55%)	30 (50%)
ASA					
3	*n* (%)	12 (63%)	13 (62%)	11 (55%)	36 (60%)
4	*n* (%)	7 (37%)	8 (38%)	9 (45%)	24 (40%)
CPB time (min)	Mean (SD)	125.2 (60.8)	151.1 (54.3)	111.7 (49.3)	129.8 (56.5)
	Median [IQR]	111.0 [82.5, 162.0]	154.0 [117.0, 177.0]	118.5 [78.0, 127.2]	122.5 [88.0, 163.5]
Aortic cross‐clamp time (min)	Mean (SD)	70.3 (40.0)	95.7 (42.3)	63.1 (37.5)	76.8 (41.9)
	Median [IQR]	57.0 [47.5, 83.5]	94.0 [64.0, 126.0]	72.0 [25.2, 85.8]	75.0 [48.8, 98.0]
Duration of surgery (Incision‐to‐suture time) (min)	Mean (SD)	265.9 (106.9)	282.5 (78.1)	273.5 (114.6)	274.2 (99.2)
	Median [IQR]	241.0 [185.0, 283.0]	276.0 [226.0, 365.0]	227.5 [197.2, 353.2]	251.0 [191.5, 335.5]
Type of surgery					
Aortic valve repair	*n* (%)	1 (5%)	2 (10%)	0 (0%)	3 (5%)
Atrial septal defect	*n* (%)	2 (11%)	2 (10%)	4 (20%)	8 (13%)
Atrioventricular septal defect	*n* (%)	2 (11%)	5 (24%)	3 (15%)	10 (17%)
Complex corrective surgery	*n* (%)	1 (5%)	0 (0%)	0 (0%)	1 (2%)
Glenn procedure with arch reconstruction	*n* (%)	1 (5%)	0 (0%)	0 (0%)	1 (2%)
Mitral valve repair	*n* (%)	0 (0%)	1 (5%)	1 (5%)	2 (3%)
Reconstruction of partial anomalous pulmonary venous return (right/left)	*n* (%)	1 (5%)	1 (5%)	1 (5%)	3 (5%)
Right ventricular outflow tract obstruction	*n* (%)	1 (5%)	0 (0%)	2 (10%)	3 (5%)
Ross procedure	*n* (%)	0 (0%)	0 (0%)	1 (5%)	1 (2%)
Tetralogy of Fallot	*n* (%)	4 (21%)	4 (19%)	3 (15%)	11 (18%)
Ventricular septal defect	*n* (%)	6 (32%)	6 (29%)	5 (25%)	17 (28%)

*Note:* Absolute frequencies are denoted by *n*.

Abbreviations: ASA, American Society of Anesthesiologists; CPAP 5, continuous positive airway pressure at 5 mbar; CPB, cardiopulmonary bypass; IQR, interquartile range; LPV, lung‐protective ventilation; SD, standard deviation.

### 3.2 Global Respiratory Mechanics

Table 2 presents the global respiratory mechanics during controlled mechanical ventilation performed preoperatively and postoperatively. For a more comprehensive view of the results from statistical analysis, refer to Table S2. PEEP (mbar), PIP (mbar), and driving pressure (mbar) were comparable between all groups. PEEP remained stable preoperatively and postoperatively. In contrast, mean PIP and driving pressure increased statistically significantly over time (Table 2). Mean dynamic compliance (Cdyn; Pa L^−1^) decreased statistically significantly over time. The highest mean reduction in dynamic compliance was observed in the group receiving apnea, followed by the group receiving CPAP at 5 mbar and the group receiving LPV. MP increased slightly but statistically significantly over time. No statistically significant differences were observed between groups, nor was there a statistically significant interaction between time and group.

**TABLE 2:** Global respiratory mechanics during pre‐op (preoperative) and post‐op (postoperative) controlled mechanical ventilation (CMV), categorized into three intervention groups.

			Total		Apnea		CPAP 5		LPV		*p*‐values, *F*‐tests			
Parameter	Time	Measure	*n*		*n*		*n*		*n*		Age	Time	Group	Time × group
PIP (mbar)	Pre‐op CMV	Mean (SD)	60	16.7 (2.7)	19	17.2 (3.2)	21	16.4 (2.4)	20	16.6 (2.5)	**< 0.001**	**< 0.001**	0.304	0.452
		Median [IQR]	60	16.0 [14.1, 18.7]	19	16.0 [14.4, 18.9]	21	16.0 [14.0, 18.0]	20	16.0 [14.8, 18.7]				
	Post‐op CMV	Mean (SD)	58	17.8 (2.8)	19	18.7 (3.1)	19	17.5 (2.6)	20	17.3 (2.7)				
		Median [IQR]	58	17.5 [15.7, 19.0]	19	17.8 [16.5, 20.9]	19	17.0 [15.6, 18.5]	20	17.5 [15.0, 19.0]				
PEEP (mbar)	Pre‐op CMV	Mean (SD)	60	4.9 (0.9)	19	5.2 (1.2)	21	4.7 (0.7)	20	4.8 (0.6)	0.842	0.141	0.17	0.777
		Median [IQR]	60	5.0 [5.0, 5.0]	19	5.0 [5.0, 5.0]	21	5.0 [5.0, 5.0]	20	5.0 [5.0, 5.0]				
	Post‐op CMV	Mean (SD)	58	4.9 (0.9)	19	5.3 (1.2)	19	4.7 (0.8)	20	4.8 (0.6)				
		Median [IQR]	58	5.0 [5.0, 5.0]	19	5.0 [5.0, 5.0]	19	5.0 [5.0, 5.0]	20	5.0 [5.0, 5.0]				
PIP‐PEEP (mbar)	Pre‐op CMV	Mean (SD)	60	11.8 (2.6)	19	12.0 (2.7)	21	11.7 (2.7)	20	11.8 (2.6)	**< 0.001**	**< 0.001**	0.674	0.476
		Median [IQR]	60	11.3 [9.7, 13.9]	19	11.7 [9.4, 13.9]	21	12.0 [9.0, 13.0]	20	11.0 [10.0, 13.9]				
	Post‐op CMV	Mean (SD)	58	12.9 (2.6)	19	13.5 (2.5)	19	12.7 (2.7)	20	12.5 (2.7)				
		Median [IQR]	58	12.6 [10.8, 14.8]	19	12.9 [11.9, 15.3]	19	12.0 [10.7, 14.5]	20	12.5 [10.0, 14.0]				
Cdyn (Pa L^−1^)	Pre‐op CMV	Mean (SD)	60	9.9 (7.3)	19	9.0 (7.3)	21	9.0 (5.8)	20	11.7 (8.6)	**< 0.001**	**0.0325**	0.282	0.629
		Median [IQR]	60	6.5 [5.0, 14.7]	19	5.4 [4.8, 10.7]	21	6.9 [5.3, 10.1]	20	9.4 [5.4, 15.1]				
	Post‐op CMV	Mean (SD)	56	8.5 (7.4)	18	6.9 (4.3)	19	7.1 (5.0)	19	11.4 (10.6)				
		Median [IQR]	56	5.7 [4.7, 10.5]	18	5.5 [4.4, 8.0]	19	5.6 [4.6, 6.6]	19	9.1 [5.4, 12.9]				
MP (J min^−1^ kg^−1^)	Pre‐op CMV	Mean (SD)	60	0.4 (0.1)	19	0.4 (0.1)	21	0.4 (0.1)	20	0.4 (0.1)	**< 0.001**	**0.0374**	0.755	0.481
		Median [IQR]	60	0.4 [0.3, 0.5]	19	0.4 [0.3, 0.4]	21	0.4 [0.3, 0.5]	20	0.4 [0.3, 0.4]				
	Post‐op CMV	Mean (SD)	58	0.4 (0.2)	19	0.4 (0.2)	19	0.4 (0.1)	20	0.4 (0.1)				
		Median [IQR]	58	0.4 [0.3, 0.5]	19	0.4 [0.3, 0.5]	19	0.4 [0.3, 0.5]	20	0.4 [0.3, 0.5]				

*Note:* Measured parameters include positive end‐expiratory pressure (PEEP; mbar), peak inspiratory pressure (PIP; mbar), driving pressure (PIP–PEEP; mbar), dynamic compliance (Cdyn; Pa L^−1^), and mechanical power (MP; J min^−1^ kg^−1^). The last column displays *p*‐values from global *F*‐tests in linear mixed‐effects models, with values less than 0.05 shown in bold. Time × Group indicates the interaction between time and group. Absolute frequencies are denoted by *n*.

Abbreviations: CPAP 5, continuous positive airway pressure at 5 mbar; IQR, interquartile range; LPV, lung‐protective ventilation; SD, standard deviation.

Intraoperative LPV (group 3) resulted in an individualized driving pressure of 3.3 (0.5) mbar and a PIP of 8.3 (0.5) mbar.

### 3.3 Regional Analysis of EIT Data

**FIGURE 2:**
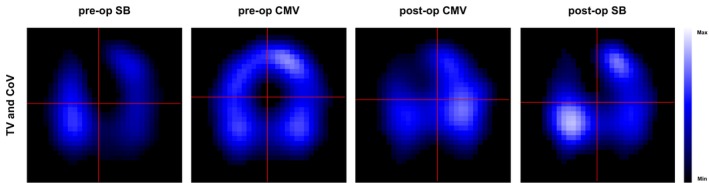
Functional Electrical Impedance Tomography (EIT) images. Representative measurements during spontaneous breathing preoperatively (pre‐op SB), controlled mechanical ventilation preoperatively (pre‐op CMV), controlled mechanical ventilation postoperatively (post‐op CMV), and spontaneous breathing postoperatively (post‐op SB). The images display the relative regional distribution of tidal variation (TV) as a surrogate for distribution of tidal volume; the red lines indicate the Center of Ventilation (CoV) in horizontal (CoVx) and vertical (CoVy) directions.

**FIGURE 3:**
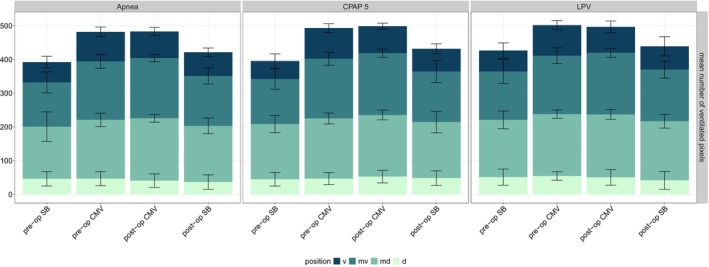
Regional analysis of Electrical Impedance Tomography (EIT) data, showing the distribution of ventilated pixels as a surrogate for ventilated lung volume (in absolute number of pixels) across four equidistant gravitational regions of interest (ROIs): Ventral (v), mid‐ventral (mv), mid‐dorsal (md), and dorsal (d). Measurements were obtained during preoperative (pre‐op) and postoperative (post‐op) spontaneous breathing (SB) and controlled mechanical ventilation (CMV) in three intervention groups. Linear mixed‐effects models revealed a statistically significant effect of ROI, time, and their interaction on the number of ventilated pixels. The colored bars indicate the mean values, and the error bars represent one standard deviation. CPAP 5, continuous positive airway pressure at 5 mbar; LPV, lung‐protective ventilation.

Representative images from EIT are given in Figure 2. The number of ventilated pixels changed statistically significantly over time and across different ROIs (Figure 3), with detailed values from statistical analysis provided in Table S3. The statistically significant interaction between ROIs and time (Table S3) suggests that the pattern of change in ventilated pixels over time varied depending on the specific ROI. Compared to spontaneous breathing performed preoperatively, mechanical ventilation caused a statistically significant increase in ventilated pixels (Figure 3; Table S3). This increase initially occurred in the mid‐dorsal region, subsequently progressing to the region that was mid‐ventral. Following emergence from anesthesia, termination of controlled mechanical ventilation normalized the number of ventilated pixels to baseline values (Figure 3). No statistically significant effect of the mode of ventilation performed during surgery while cross‐clamping of the aorta was observed on this parameter (Figure 3; Table S3).

### 3.4 Global Parameters Related to Ventilation Distribution and Inhomogeneity

**FIGURE 4:**
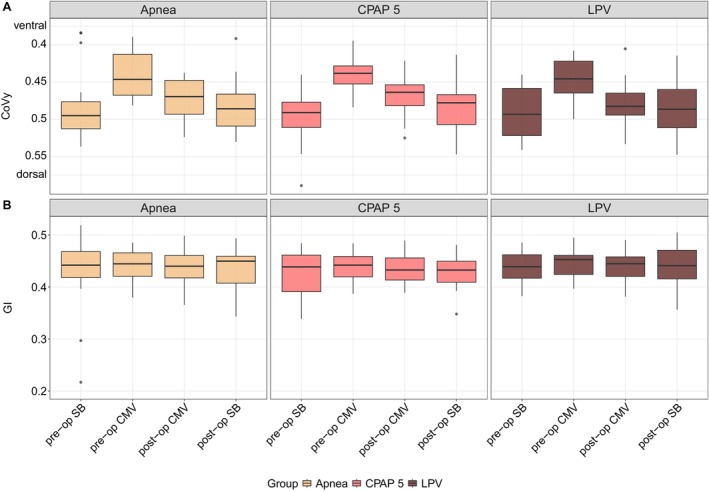
Global ventilation distribution and inhomogeneity parameters represented as boxplots derived from Electrical Impedance Tomography (EIT) scans during preoperative (pre‐op) and postoperative (post‐op) spontaneous breathing (SB) and controlled mechanical ventilation (CMV) categorized into three intervention groups. (A) Center of Ventilation in the gravity‐dependent direction (CoVy), where a CoVy of 0 indicates a most ventral, a CoVy of 1 a most dorsal distribution of tidal volume. (B) Global Inhomogeneity (GI) Index, where a GI of 1 indicates a most inhomogeneous, a GI of 0 a most homogeneous distribution of tidal volume within the image. CPAP 5, continuous positive airway pressure at 5 mbar; LPV, lung‐protective ventilation.

Consistent findings were obtained from global parameters based on EIT describing ventilation distribution and inhomogeneity (Figure 4). The Center of Ventilation (CoV) along the axis from ventral to dorsal shifted statistically significantly toward the ventral lung upon initiation of controlled mechanical ventilation performed preoperatively (Figure 4A), as detailed in Table S4. This effect was less pronounced during mechanical ventilation performed postoperatively. Following emergence from anesthesia, spontaneous breathing restored baseline values. The Global Inhomogeneity (GI) Index showed no statistically significant changes in tidal volume image homogeneity over time (Figure 4B; Table S4). No statistically significant effect was observed from different modes of ventilation performed during surgery while aortic cross‐clamping on these parameters (Figure 4; Table S4).

### 3.5 Blood Sample Analysis

The results of blood sample analysis are presented in Tables S5 and S6. The groups demonstrate a high variability in inflammatory marker levels at baseline. Baseline correction was performed in the linear mixed‐effects models. Interleukin‐6 was the only biomarker measured demonstrating a statistically significant effect over time, indicating a systematic response to inflammation in all three groups receiving intervention from preoperatively to 48 h postoperatively. All other biomarkers, like RAGE, SP‐D, or Ang‐2/Ang‐1 ratio, showed no statistically significant effect over time, effect between groups, or interaction between time and group.

### 3.6 Feasibility of LPV

LPV during aortic cross‐clamping was successfully implemented in nearly all patients without compromising surgical conditions. Respiratory movements associated with LPV did not interfere with surgical procedures. One patient required the reduction of PIP from 7 to 5 mbar due to surgeon preference regarding minimal lung‐dependent respiratory movements in the surgical field. Following this pressure adjustment, surgical conditions were optimized and the procedure continued without further impairment. The PEEP remained unchanged.

### 3.7 Arterial Blood Gas Analysis

Results from arterial blood gas analysis performed preoperatively and postoperatively are presented in Tables S7 and S8.

## 4 Discussion

This pilot study aimed to evaluate the impact of different ventilation strategies during aortic cross‐clamping on postoperative ventilation inhomogeneity, as well as on pulmonary and systemic inflammation in pediatric patients with CHD undergoing CPB for cardiac surgery. Additionally, the feasibility of applying LPV during aortic cross‐clamping and open‐heart surgery was assessed.

A mild impairment of global respiratory mechanics was observed postoperatively, likely reflecting the effects of open‐heart surgery and mechanical ventilation, independent of the ventilation strategy used. EIT revealed an increase in lung volume and ventilation redistribution from dorsal to ventral regions during the transition from spontaneous breathing to mechanical ventilation, consistent with the well‐established phenomenon of ventral shift under these conditions and thereby supporting the validity of our measurements [21]. However, the hypothesis that this redistribution during mechanical ventilation results in persistent dorsal atelectasis was not confirmed by our results. Postoperatively, lung aeration returned to preoperative state without significant residual atelectasis. The ventilatory strategies including LPV did not affect the distribution of ventilation postoperatively, which might be partially explained by the routine use of recruitment maneuvers at the end of CPB.

Recruitment maneuvers following general anesthesia and CPB have been shown to consistently improve respiratory mechanics and arterial oxygenation in various settings [22], including pediatric cardiac surgery where lung ultrasound‐guided recruitment maneuvers reduced postoperative desaturation events and ventilation time [23]. The lack of intergroup differences in respiratory and inflammatory variables in our study likely reflects the standardized recruitment maneuvers performed at the end of CPB. These maneuvers, routinely applied to de‐air cardiac cavities and re‐expand collapsed lung tissue, may have provided comparable pulmonary benefits across all groups, thereby masking potential effects of the intraoperative ventilation strategies.

The use of CPB in cardiac surgery is widely known to increase inflammation [4, 24]. Our findings indicate that endothelial barrier disruption and inflammatory responses are primarily associated with the initiation of CPB and the surgical procedure itself, rather than with different ventilatory strategies, as this inflammatory reaction substantially resolves within 48 h postoperatively in all groups. The absence of statistically significant changes in lung‐specific markers RAGE and SP‐D across all groups and over time again suggests that recruitment maneuvers alone may sufficiently mitigate lung injury during aortic cross‐clamping, regardless of the ventilation strategy used.

LPV was successfully applied in nearly all patients without affecting surgical conditions; only one case required minor PIP adjustment to accommodate surgical preferences. These findings suggest that LPV can be used during aortic cross‐clamping without interfering with surgical procedures and extend previous research [25] by demonstrating its feasibility during active surgical phases in pediatric patients. However, the sample size of this pilot study is too small to draw definitive conclusions regarding the safety of LPV in this setting.

While statistically significant differences were observed in blood gas parameters, these differences are not deemed clinically relevant.

Our hypothesis that LPV during CPB would reduce both systemic and pulmonary inflammatory responses and improve respiratory function postoperatively could not be supported by our data. No statistically significant differences were observed between groups regarding postoperative respiratory function and inflammatory status.

Notably, ventilation distribution and respiratory variables were not measured at the end of CPB before performing the recruitment maneuver. Thus, we cannot rule out that the different ventilatory strategies may have resulted in significant changes in ventilation distribution, respiratory mechanics, or gas exchange at this phase of anesthesia, which were subsequently masked by the recruitment maneuvers.

Further limitations include the small sample size, as of 68 enrolled patients, 8 were excluded (Figure S1), leaving only 19–21 per group, which may impact the study's conclusions. Given the limited sample size and the generally small observed effect sizes, corresponding confidence intervals were imprecise, leading to a wide and clinically challenging range of required sample sizes (Supporting Information, p. 20), limiting their use for reliable sample size estimation and emphasizing the need to complement such estimates with clinical expertise and prior evidence. Randomization may also not have fully balanced baseline characteristics. Nevertheless, potential age differences were explicitly accounted for in the analyses.

The timing of the 6 h postoperative blood sample varied due to collections late at night.

The cohort showed heterogeneous pulmonary physiology, particularly regarding differences in pulmonary blood flow. The variability in disease stage and patient characteristics, combined with the small sample size, did not allow for meaningful subgroup analyses.

Additionally, driving pressure during LPV was set to 20% of pre‐clamp PIP to individualize intraoperative settings according to age‐dependent lung mechanics. Although this approach was applied consistently within our protocol, it remains arbitrary due to the absence of evidence on which ventilation settings can be safely tolerated without impairing surgical conditions. To our knowledge, no data or surveys exist on current practices regarding ventilation during this period across pediatric cardiac centers, and substantial variability between institutions is likely, underscoring the pilot character of our study and the need for collaborative efforts to define standardized strategies for lung protection during cross‐clamping.

### 4.1 Future Studies

Further studies should incorporate EIT‐guided monitoring of recruitment maneuvers at the end of CPB, with measurements obtained before and after recruitment to clarify whether benefits observed postoperatively are primarily attributable to end‐of‐bypass recruitment protocols or can additionally be gained from optimized ventilation during cross‐clamping. Intraoperative EIT monitoring during CPB could provide real‐time assessments of ventilatory strategies and their immediate pulmonary effects. However, existing EIT electrode belts are designed to encircle the intact thorax and do not work during open‐chest procedures. Technological advances enabling EIT monitoring during sternotomy would substantially improve understanding of intraoperative lung mechanics. This pilot study aims to stimulate discussion on optimal ventilation strategies during aortic cross‐clamping in pediatric cardiac surgery and foster collaborative research efforts toward evidence‐based, standardized approaches for lung protection during this critical surgical phase.

## 5 Conclusion

Our findings indicate that the ventilation strategy during aortic cross‐clamping in CPB in pediatric patients does not relevantly impact postoperative respiratory mechanics, ventilation inhomogeneity, or pulmonary and systemic inflammatory responses when recruitment maneuvers are performed at the end of CPB. LPV during aortic cross‐clamping was feasible without interfering with cardiac surgery procedures by disturbing surgeons through lung‐dependent movements. Larger studies using advanced imaging are needed to assess whether intraoperative ventilation provides additional benefit beyond post‐bypass recruitment to prevent atelectasis formation and mitigate inflammatory injury.

## Author Contributions

Charlotte Billstein: Patient recruitment, data acquisition, formal analysis, drafting the first version of the manuscript, reviewing, and editing. Alina Schenk: Statistical analysis, writing, reviewing, and editing. Mathieu Vergnat: Conceptualization, reviewing, editing, and management of study participants. Patrick Jakobs: Patient recruitment and data acquisition. Stilla Frede: Conceptualization, reviewing, and editing. Christian P. Putensen: Conceptualization, supervision, reviewing, and editing. Thomas Muders: Conceptualization, methodology, formal analysis, supervision, EIT software, writing, reviewing, and editing. Ehrenfried Schindler: Conceptualization, methodology, formal analysis, supervision, project administration, revising, reviewing, and editing.

## Disclosure

Declaration of Generative AI and AI‐Assisted Technologies in the Writing Process: During the preparation of this work, the authors used ChatGPT and Claude to improve readability and language. After using this tool, the authors reviewed and edited the content as needed and took full responsibility for the content of the publication.

## Conflicts of Interest

The authors declare no conflicts of interest.

## Supporting information


**Table S1:** Study population—left‐to‐right shunt.
**Table S2:** Global respiratory mechanics—detailed results.
**Table S3:** Regional analysis of EIT data—number of ventilated pixels.
**Table S4:** Global ventilation distribution and inhomogeneity parameters.
**Table S5:** Venous blood sample analysis at four time points: preoperatively (pre‐op), 6 h postoperatively (6 h post‐op), 24 h postoperatively (24 h post‐op), and 48 h postoperatively (48 h post‐op), across three intervention groups. Biomarker concentrations were measured using a multiplex immunoassay, including interleukin‐6 (IL‐6), angiopoietin‐2 (Ang‐2), receptor for advanced glycation end‐products (RAGE), surfactant protein D (SP‐D), angiopoietin‐1 (Ang‐1), and the angiopoietin‐2/angiopoietin‐1 ratio (Ang‐2/Ang‐1). All parameters are expressed in pg mL^−1^ and underwent log_10_ transformation for analysis, except for the Ang‐2/Ang‐1 ratio which remained untransformed. The last column displays *p*‐values from global *F*‐tests in linear mixed‐effects models, with values less than 0.05 shown in bold. CPAP 5, continuous positive airway pressure at 5 mbar; IQR, interquartile range; LPV, lung‐protective ventilation; SD, standard deviation. Time × Group indicates the interaction between time and group. Time × Baseline indicates the interaction between time and baseline. Absolute frequencies are denoted by *n*.
**Table S6:** Detailed results of blood sample analysis.
**Table S7:** Arterial blood gas analysis performed preoperatively (pre‐op) and postoperatively (post‐op) across three intervention groups. Measured parameters include pH (mol L^−1^), partial pressure of carbon dioxide (paCO_2_; mmHg), partial pressure of oxygen (paO_2_; mmHg), arterial oxygen saturation (SaO_2_; %), base excess (BE; mmol L^−1^) and lactate (mmol L^−1^). The last column displays *p* values from global *F*‐tests in linear mixed‐effects models, with values less than 0.05 shown in bold. CPAP 5, continuous positive airway pressure at 5 mbar; IQR, interquartile range; LPV, lung‐protective ventilation; SD, standard deviation. Time × Group indicates the interaction between time and group. Absolute frequencies are denoted by *n*.
**Table S8:** Arterial blood gas analysis—statistical results.
**Figure S1:** CONSORT flowchart. CPAP 5, continuous positive airway pressure at 5 mbar; ICU, intensive care unit; LPV, lung‐protective ventilation. Absolute frequencies are denoted by n.

## Data Availability

The data that support the findings of this study are available from the corresponding author upon reasonable request.
