# Supplementary material for: Effects of Intraoperative Ventilation Strategies on Ventilation Inhomogeneity and Inflammatory Response in Pediatric Cardiac Surgery—A Randomized Pilot Study
Source: Paediatr Anaesth. 2025 Oct 23;36(1):88–99. doi: 10.1111/pan.70066 (PMC12686771; doi:10.1111/pan.70066)
Supplement: Supplementary file 1 — Table S1: Study population—left‐to‐right shunt. Table S2: Global respiratory mechanics—detailed results. Table S3: Regional analysis of EIT data—number of ventilated pixels. Table S4: Global ventilation distribution and inhomogeneity parameters. Table S5: Venous blood sample analysis at four time points: preoperatively (pre‐op), 6 h postoperatively (6 h post‐op), 24 h postoperatively (24 h post‐op), and 48 h postoperatively (48 h post‐op), across three intervention groups. Biomarker concentrations were measured using a multiplex immunoassay, including interleukin‐6 (IL‐6), angiopoietin‐2 (Ang‐2), receptor for advanced glycation end‐products (RAGE), surfactant protein D (SP‐D), angiopoietin‐1 (Ang‐1), and the angiopoietin‐2/angiopoietin‐1 ratio (Ang‐2/Ang‐1). All parameters are expressed in pg mL−1 and underwent log10 transformation for analysis, except for the Ang‐2/Ang‐1 ratio which remained untransformed. The last column displays p‐values from global F‐tests in linear mixed‐effects models, with values less than 0.05 shown in bold. CPAP 5, continuous positive airway pressure at 5 mbar; IQR, interquartile range; LPV, lung‐protective ventilation; SD, standard deviation. Time × Group indicates the interaction between time and group. Time × Baseline indicates the interaction between time and baseline. Absolute frequencies are denoted by n. Table S6: Detailed results of blood sample analysis. Table S7: Arterial blood gas analysis performed preoperatively (pre‐op) and postoperatively (post‐op) across three intervention groups. Measured parameters include pH (mol L−1), partial pressure of carbon dioxide (paCO2; mmHg), partial pressure of oxygen (paO2; mmHg), arterial oxygen saturation (SaO2; %), base excess (BE; mmol L−1) and lactate (mmol L−1). The last column displays p values from global F‐tests in linear mixed‐effects models, with values less than 0.05 shown in bold. CPAP 5, continuous positive airway pressure at 5 mbar; IQR, interquartile range; LPV, lung‐protective [file PAN-36-88-s001.docx]

**SUPPLEMENTARY MATERIAL**

**METHODS – Blood sample collection and multiplex assay analysis**

To evaluate inflammation, this study examined the progression of inflammatory markers interleukin-6 (IL-6) and angiopoietins. Angiopoietin-1 (Ang-1) is known for mitigating the cellular inflammatory response and protective effects on the vasculature by preventing vascular leakage.^1,2^ Conversely, angiopoietin-2 (Ang-2) is linked to inflammatory markers, promotes inflammation and increases capillary permeability, contributing to capillary leak syndrome.^2,3,4,5^ Recent studies indicate that the Ang-2/Ang-1 ratio is a reliable marker for the severity of capillary leak syndrome.^5^

To evaluate lung damage, this study analyzed the plasma receptor for advanced glycation end products (RAGE) and surfactant protein D (SP-D). RAGE is a pattern recognition receptor in the immunoglobulin family. Produced mainly by alveolar epithelial type I cells, RAGE is secreted into the alveolar space after cellular damage and enters the bloodstream as a potential lung injury biomarker.^6,7^ SP-D, a pattern recognition molecule from the collectin family, is secreted by alveolar epithelial type II cells. SP-D serves as a marker for alveolar epithelial damage, with elevated plasma linked to severe pediatric acute respiratory distress syndrome (PARDS).^8,9^

**References**

[1] Brindle NPJ, Saharinen P, Alitalo K. Signaling and Functions of Angiopoietin-1 in Vascular Protection. Circulation Research 2006;98:1014–23. https://doi.org/10.1161/01.RES.0000218275.54089.12.

[2] Giuliano JS, Lahni PM, Bigham MT, Manning PB, Nelson DP, Wong HR, et al. Plasma angiopoietin-2 levels increase in children following cardiopulmonary bypass. Intensive Care Med 2008;34:1851–7. https://doi.org/10.1007/s00134-008-1174-9.

[3] Schuldt EA, Lieb W, Dörr M, Lerch MM, Völzke H, Nauck M, et al. Circulating angiopoietin-2 and its soluble receptor Tie-2 concentrations are related to inflammatory markers in the general population. Cytokine 2018;105:1–7. https://doi.org/10.1016/j.cyto.2018.02.003.

[4] Parikh SM, Mammoto T, Schultz A, Yuan H-T, Christiani D, Karumanchi SA, et al. Excess Circulating Angiopoietin-2 May Contribute to Pulmonary Vascular Leak in Sepsis in Humans. PLoS Med 2006;3:e46. https://doi.org/10.1371/journal.pmed.0030046.

[5] Heuberger D, Wendel-Garcia PD, Sazpinar O, Müller M, Klein H, Kim B-S, et al. The Angiopoietin-2/Angiopoietin-1 ratio increases early in burn patients and predicts mortality. Cytokine 2023;169:156266. https://doi.org/10.1016/j.cyto.2023.156266.

[6] Griffiths MJD, McAuley DF. RAGE: a biomarker for acute lung injury. Thorax 2008;63:1034–6. https://doi.org/10.1136/thx.2008.101493.

[7] Agostoni P, Banfi C, Brioschi M, Magrì D, Sciomer S, Berna G, et al. Surfactant protein B and RAGE increases in the plasma during cardiopulmonary bypass: a pilot study. Eur Respir J 2011;37:841–7. https://doi.org/10.1183/09031936.00045910.

[8] Dahmer MK, Flori H, Sapru A, Kohne J, Weeks HM, Curley MAQ, et al. Surfactant Protein D Is Associated With Severe Pediatric ARDS, Prolonged Ventilation, and Death in Children With Acute Respiratory Failure. Chest 2020;158:1027–35. https://doi.org/10.1016/j.chest.2020.03.041.

[9] Sorensen GL. Surfactant Protein D in Respiratory and Non-Respiratory Diseases. Front Med 2018;5:18. https://doi.org/10.3389/fmed.2018.00018.

**RESULTS**

**Supplementary Table S1: Study population – Left-to-right shunt**

| **Parameter** |  | **Measure** | **Apnea**  **(n = 19)** | **CPAP 5**  **(n = 21)** | **LPV**  **(n = 20)** | **Total**  **(n = 60)** |
| --- | --- | --- | --- | --- | --- | --- |
| Left-to-right shunt |  | n (%) | 12 (63.2%) | 14 (66.7%) | 13 (65.0%) | 39 (65.0%) |

**Supplementary Table S2: Global respiratory mechanics – Detailed results**

|  | **PIP (mbar)** | | | **PEEP (mbar)** | | |
| --- | --- | --- | --- | --- | --- | --- |
| *Predictors* | *Estimates* | *CI* | *p* | *Estimates* | *CI* | *p* |
| Intercept | 18.416 | 17.162 – 19.670 | **<0.001** | 5.233 | 4.772 – 5.695 | **<0.001** |
| Age [months] | -0.066 | -0.096 – -0.036 | **<0.001** | -0.001 | -0.013 – 0.011 | 0.841 |
| Time [post-op CMV] | 1.558 | 0.564 – 2.552 | **0.002** | 0.047 | -0.039 – 0.134 | 0.279 |
| Group [CPAP 5] | -0.876 | -2.420 – 0.667 | 0.263 | -0.498 | -1.053 – 0.057 | 0.078 |
| Group [LPV] | -0.218 | -1.786 – 1.351 | 0.784 | -0.412 | -0.976 – 0.153 | 0.151 |
| Time x Group [post-op CMV x CPAP 5] | -0.421 | -1.819 – 0.978 | 0.552 | 0.005 | -0.117 – 0.127 | 0.941 |
| Time x Group [post-op CMV x LPV] | -0.888 | -2.275 – 0.500 | 0.207 | -0.035 | -0.155 – 0.086 | 0.568 |
| **Random Effects** | | | | | | |
| σ^2^ | 2.39 | | | 0.02 | | |
| τ_00_ | 3.66 _id_ | | | 0.76 _id_ | | |
| Observations | 118 | | | 118 | | |
| Marginal R^2^ / Conditional R^2^ | 0.261 / 0.708 | | | 0.059 / 0.978 | | |

|  | **PIP-PEEP (mbar)** | | | **Cdyn (Pa L^-1^)** | | |
| --- | --- | --- | --- | --- | --- | --- |
| *Predictors* | *Estimates* | *CI* | *p* | *Estimates* | *CI* | *p* |
| Intercept | 13.184 | 11.989 – 14.379 | **<0.001** | 3.998 | 1.356 – 6.641 | **0.003** |
| Age [months] | -0.065 | -0.093 – -0.037 | **<0.001** | 0.263 | 0.202 – 0.323 | **<0.001** |
| Time [post-op CMV] | 1.511 | 0.523 – 2.498 | **0.003** | -2.200 | -4.839 – 0.439 | 0.101 |
| Group [CPAP 5] | -0.378 | -1.852 – 1.095 | 0.612 | 0.456 | -2.835 – 3.747 | 0.784 |
| Group [LPV] | 0.194 | -1.304 – 1.692 | 0.798 | 1.400 | -1.943 – 4.744 | 0.408 |
| Time x Group [post-op CMV x CPAP 5] | -0.414 | -1.803 – 0.976 | 0.556 | 0.071 | -3.600 – 3.741 | 0.970 |
| Time x Group [post-op CMV x LPV] | -0.853 | -2.232 – 0.526 | 0.223 | 1.575 | -2.108 – 5.258 | 0.399 |
| **Random Effects** | | | | | | |
| σ^2^ | 2.36 | | | 16.20 | | |
| τ_00_ | 3.15 _id_ | | | 11.27 _id_ | | |
| Observations | 118 | | | 116 | | |
| Marginal R^2^ / Conditional R^2^ | 0.256 / 0.681 | | | 0.507 / 0.709 | | |

|  | **MP (J min^-1^ kg^-1^)** | | |  |
| --- | --- | --- | --- | --- |
| *Predictors* | *Estimates* | *CI* | *p* |  |
| Intercept | 0.455 | 0.392 – 0.517 | **<0.001** |  |
| Age [months] | -0.004 | -0.005 – -0.002 | **<0.001** |  |
| Time [post-op CMV] | 0.050 | 0.001 – 0.098 | **0.045** |  |
| Group [CPAP 5] | 0.020 | -0.057 – 0.097 | 0.605 |  |
| Group [LPV] | 0.031 | -0.047 – 0.109 | 0.429 |  |
| Time x Group [post-op CMV x CPAP 5] | -0.042 | -0.110 – 0.027 | 0.228 |  |
| Time x Group [post-op CMV x LPV] | -0.018 | -0.086 – 0.050 | 0.609 |  |
| **Random Effects** | | | | |
| σ^2^ | 0.01 | | |  |
| τ_00_ | 0.01 _id_ | | |  |
| Observations | 118 | | |  |
| Marginal R^2^ / Conditional R^2^ | 0.266 / 0.719 | | |  |

**Supplementary Table S3: Regional analysis of EIT data – Number of ventilated pixels**

| **Mean (SD) – Ventilated pixels** | | | | |
| --- | --- | --- | --- | --- |
| **Group** | **pre-op SB** | **pre-op CMV** | **post-op CMV** | **post-op SB** |
| Total | 408.2 (77.2) | 495.1 (44.9) | 493.0 (38.3) | 433.9 (72.6) |
| Apnea | 402.8 (76.8) | 489.9 (50.8) | 482.9 (37.5) | 429.4 (45.9) |
| CPAP 5 | 395.4 (71.9) | 493.1 (44.9) | 498.6 (31.4) | 432.0 (84.1) |
| LPV | 426.4 (83.0) | 501.6 (40.9) | 496.6 (45.1) | 438.9 (82.4) |

|  | **Ventilated pixels (absolute number)** | | |  |
| --- | --- | --- | --- | --- |
| *Predictors* | *Estimates* | *CI* | *p* | *p (F-test)* |
| Intercept | 53.02 | 44.86 – 61.18 | **<0.001** | **-** |
| Age [months] | 0.27 | 0.14 – 0.40 | **<0.001** | **<0.001** |
| Group [CPAP 5] | -2.25 | -11.77 – 7.26 | 0.642 | 0.350 |
| Group [LPV] | 2.57 | -7.05 – 12.20 | 0.600 |  |
| Time [pre-op CMV] | 31.91 | 23.33 – 40.49 | **<0.001** | **<0.001** |
| Time [post-op CMV] | 21.03 | 12.47 – 29.60 | **<0.001** |  |
| Time [post-op SB] | 9.39 | 0.24 – 18.54 | **0.044** |  |
| ROI [mv] | 74.44 | 65.80 – 83.07 | **<0.001** | **<0.001** |
| ROI [md] | 100.33 | 91.69 – 108.96 | **<0.001** |  |
| ROI [d] | -16.55 | -25.28 – -7.83 | **<0.001** |  |
| Time x ROI [pre-op CMV x mv] | 7.02 | -2.74 – 16.77 | 0.158 | **<0.001** |
| Time x ROI [post-op CMV x mv] | 25.27 | 15.51 – 35.02 | **<0.001** |  |
| Time x ROI [post-op SB x mv] | 4.08 | -6.21 – 14.36 | 0.437 |  |
| Time x ROI [pre-op CMV x md] | -15.45 | -25.20 – -5.70 | **0.002** |  |
| Time x ROI [post-op CMV x md] | 1.58 | -8.17 – 11.34 | 0.750 |  |
| Time x ROI [post-op SB x md] | -3.93 | -14.22 – 6.36 | 0.453 |  |
| Time x ROI [pre-op CMV x d] | -29.40 | -39.22 – -19.58 | **<0.001** |  |
| Time x ROI [post-op CMV x d] | -18.81 | -28.58 – -9.03 | **<0.001** |  |
| Time x ROI [post-op SB x d] | -15.60 | -25.98 – -5.23 | **0.003** |  |
| Group x Time [CPAP 5 x pre-op CMV] | 1.98 | -6.53 – 10.48 | 0.648 | 0.653 |
| Group x Time [LPV x pre-op CMV] | -3.65 | -12.25 – 4.95 | 0.405 |  |
| Group x Time [CPAP 5 x post-op CMV] | 2.75 | -5.72 – 11.23 | 0.524 |  |
| Group x Time [LPV x post-op CMV] | -5.50 | -14.07 – 3.08 | 0.209 |  |
| Group x Time [CPAP 5 x pre-op SB] | 2.53 | -6.70 – 11.76 | 0.591 |  |
| Group x Time [LPV x pre-op SB] | -3.45 | -12.53 – 5.63 | 0.456 |  |
| Group x ROI [CPAP 5 x mv] | 4.32 | -4.38 – 13.02 | 0.330 | 0.536 |
| Group x ROI [LPV x mv] | 4.65 | -4.08 – 13.38 | 0.296 |  |
| Group x ROI [CPAP 5 x md] | 3.79 | -4.91 – 12.49 | 0.393 |  |
| Group x ROI [LPV x md] | 7.89 | -0.84 – 16.63 | 0.076 |  |
| Group x ROI [CPAP 5 x d] | 7.10 | -1.69 – 15.89 | 0.113 |  |
| Group x ROI [LPV x d] | 7.18 | -1.62 – 15.99 | 0.110 |  |
| **Random Effects** | | | |  |
| σ^2^ | 370.40 | | | |
| τ_00_ _patient_ | 67.69 | | | |
| Observations | 911 | | | |
| Marginal R^2^ / Conditional R^2^ | 0.878 / 0.897 | | | |

**Supplementary Table S4: Global ventilation distribution and inhomogeneity parameters**

| **Mean (SD) – Global Inhomogeneity Index (GI)** | | | | |
| --- | --- | --- | --- | --- |
| **Group** | **pre-op SB** | **pre-op CMV** | **post-op CMV** | **post-op SB** |
| Total | 0.432 (0.049) | 0.443 (0.028) | 0.438 (0.032) | 0.434 (0.038) |
| Apnea | 0.429 (0.070) | 0.440 (0.031) | 0.435 (0.035) | 0.431 (0.043) |
| CPAP 5 | 0.428 (0.044) | 0.440 (0.026) | 0.436 (0.030) | 0.428 (0.033) |
| LPV | 0.439 (0.029) | 0.448 (0.026) | 0.442 (0.032) | 0.443 (0.038) |

| **Mean (SD) – Center of Ventilation (CoVy)** | | | | |
| --- | --- | --- | --- | --- |
| **Group** | **pre-op SB** | **pre-op CMV** | **post-op CMV** | **post-op SB** |
| Total | 0.491 (0.039) | 0.442 (0.027) | 0.475 (0.029) | 0.485 (0.037) |
| Apnea | 0.484 (0.047) | 0.441 (0.032) | 0.474 (0.029) | 0.483 (0.037) |
| CPAP 5 | 0.497 (0.035) | 0.440 (0.022) | 0.469 (0.026) | 0.485 (0.037) |
| LPV | 0.491 (0.035) | 0.446 (0.027) | 0.481 (0.031) | 0.486 (0.038) |

|  | **CoVy** | | |  | **GI** | | |  |
| --- | --- | --- | --- | --- | --- | --- | --- | --- |
| *Predictors* | *Estimates* | *CI* | *p* | *p (F-test)* | *Estimates* | *CI* | *p* | *p (F-test)* |
| Intercept | 0.483 | 0.467 – 0.500 | **<0.001** | - | 0.427 | 0.409 – 0.445 | **<0.001** | **-** |
| Age [months] | 0.000 | -0.000 – 0.000 | 0.841 | 0.841 | 0.000 | -0.000 – 0.000 | 0.435 | 0.435 |
| Group [CPAP 5] | 0.013 | -0.008 – 0.034 | 0.211 | 0.780 | -0.001 | -0.025 – 0.023 | 0.939 | 0.371 |
| Group [LPV] | 0.007 | -0.014 – 0.028 | 0.508 |  | 0.009 | -0.015 – 0.033 | 0.461 |  |
| Time [pre-op CMV] | -0.043 | -0.059 – -0.028 | **<0.001** | **<0.001** | 0.011 | -0.012 – 0.034 | 0.357 | 0.395 |
| Time [post-op CMV] | -0.010 | -0.025 – 0.006 | 0.219 |  | 0.006 | -0.017 – 0.030 | 0.587 |  |
| Time [post-op SB] | -0.002 | -0.019 – 0.014 | 0.790 |  | 0.001 | -0.024 – 0.026 | 0.927 |  |
| Group x Time [CPAP 5 x pre-op CMV] | -0.014 | -0.035 – 0.008 | 0.204 | 0.645 | 0.002 | -0.031 – 0.034 | 0.925 | >0.999 |
| Group x Time [LPV x pre-op CMV] | -0.002 | -0.024 – 0.020 | 0.858 |  | -0.002 | -0.035 – 0.031 | 0.908 |  |
| Group x Time [CPAP 5 x post-op CMV] | -0.019 | -0.040 – 0.002 | 0.082 |  | 0.001 | -0.031 – 0.034 | 0.936 |  |
| Group x Time [LPV x post-op CMV] | -0.001 | -0.023 – 0.021 | 0.938 |  | -0.003 | -0.036 – 0.029 | 0.844 |  |
| Group x Time [CPAP 5 x pre-op SB] | -0.010 | -0.034 – 0.013 | 0.379 |  | -0.000 | -0.035 – 0.034 | 0.989 |  |
| Group x Time [LPV x pre-op SB] | -0.002 | -0.025 – 0.021 | 0.863 |  | 0.002 | -0.032 – 0.037 | 0.886 |  |
| **Random Effects** |  | | | | | | |  |
| σ^2^ | 0.00 | | | | 0.00 | | | |
| τ_00_ | 0.00 _patient_ | | | | 0.00 _patient_ | | | |
| Observations | 229 | | | | 229 | | | |
| Marginal R^2^ / Conditional R^2^ | 0.255 / 0.605 | | | | 0.028 / 0.105 | | | |

**Supplementary Table S5: Venous blood sample analysis** at four time points: preoperatively (pre-op), 6 hours postoperatively (6 h post-op), 24 hours postoperatively (24 h post-op), and 48 hours postoperatively (48 h post-op), across three intervention groups. Biomarker concentrations were measured using a multiplex immunoassay, including interleukin-6 (IL-6), angiopoietin-2 (Ang-­­2), receptor for advanced glycation end-products (RAGE), surfactant protein D (SP-D), angiopoietin-1 (Ang-1), and the angiopoietin-2/angiopoietin-1 ratio (Ang-2/Ang-1). All parameters are expressed in pg ml^-1^ and underwent log_10_ transformation for analysis, except for the Ang-2/Ang-1 ratio which remained untransformed. The last column displays p-values from global F-tests in linear mixed-effects models, with values less than 0.05 shown in bold. Abbreviations: CPAP 5 = continuous positive airway pressure at 5 mbar, IQR = interquartile range, LPV = lung-protective ventilation, SD = standard deviation. Time*Group indicates the interaction between time and group. Time*Baseline indicates the interaction between time and baseline. Absolute frequencies are denoted by n.

|  | | | **Total** | | **Apnea** | | **CPAP 5** | | **LPV** | | **p-values F-tests** | | | | | |
| --- | --- | --- | --- | --- | --- | --- | --- | --- | --- | --- | --- | --- | --- | --- | --- | --- |
| **Parameter** | **Time** | **Measure** | **n** |  | **n** |  | **n** |  | **n** |  | **Baseline** | **Age** | **Time** | **Group** | **Time* Group** | **Time* Baseline** |
| IL-6 | pre-op | Mean (SD) | 60 | 0.9 (0.2) | 19 | 0.9 (0.2) | 21 | 0.9 (0.3) | 20 | 0.8 (0.2) | **0.0446** | 0.795 | **<0.001** | 0.441 | 0.408 | 0.132 |
|  |  | Median [IQR] | 60 | 0.9 [0.7, 1.0] | 19 | 0.9 [0.7, 1.1] | 21 | 0.9 [0.7, 1.1] | 20 | 0.7 [0.6, 0.9] |  |  |  |  |  |  |
|  | 6 h post-op | Mean (SD) | 60 | 1.6 (0.4) | 19 | 1.6 (0.3) | 21 | 1.6 (0.3) | 20 | 1.5 (0.4) |  |  |  |  |  |  |
|  |  | Median [IQR] | 60 | 1.6 [1.3, 1.8] | 19 | 1.6 [1.4, 1.8] | 21 | 1.7 [1.3, 1.7] | 20 | 1.4 [1.3, 1.6] |  |  |  |  |  |  |
|  | 24 h post-op | Mean (SD) | 60 | 1.4 (0.4) | 19 | 1.6 (0.4) | 21 | 1.5 (0.3) | 20 | 1.3 (0.4) |  |  |  |  |  |  |
|  |  | Median [IQR] | 60 | 1.4 [1.2, 1.6] | 19 | 1.5 [1.4, 1.7] | 21 | 1.4 [1.3, 1.7] | 20 | 1.2 [1.0, 1.6] |  |  |  |  |  |  |
|  | 48 h post-op | Mean (SD) | 60 | 1.2 (0.3) | 19 | 1.2 (0.4) | 21 | 1.2 (0.3) | 20 | 1.1 (0.2) |  |  |  |  |  |  |
|  |  | Median [IQR] | 60 | 1.2 [1.0, 1.3] | 19 | 1.2 [1.1, 1.3] | 21 | 1.2 [1.1, 1.3] | 20 | 1.1 [0.9, 1.2] |  |  |  |  |  |  |
| Ang-2 | pre-op | Mean (SD) | 60 | 3.6 (0.3) | 19 | 3.7 (0.3) | 21 | 3.6 (0.3) | 20 | 3.7 (0.3) | **<0.001** | 0.641 | **0.0315** | 0.701 | 0.948 | 0.0811 |
|  |  | Median [IQR] | 60 | 3.7 [3.5, 3.8] | 19 | 3.7 [3.6, 3.8] | 21 | 3.7 [3.5, 3.8] | 20 | 3.6 [3.5, 3.8] |  |  |  |  |  |  |
|  | 6 h post-op | Mean (SD) | 60 | 3.9 (0.2) | 19 | 3.9 (0.2) | 21 | 3.8 (0.3) | 20 | 3.9 (0.2) |  |  |  |  |  |  |
|  |  | Median [IQR] | 60 | 3.9 [3.8, 4.0] | 19 | 3.9 [3.8, 4.0] | 21 | 3.9 [3.8, 4.0] | 20 | 3.9 [3.8, 4.0] |  |  |  |  |  |  |
|  | 24 h post-op | Mean (SD) | 60 | 3.9 (0.3) | 19 | 4.0 (0.2) | 21 | 3.8 (0.3) | 20 | 3.9 (0.3) |  |  |  |  |  |  |
|  |  | Median [IQR] | 60 | 3.9 [3.8, 4.1] | 19 | 4.0 [3.8, 4.1] | 21 | 3.9 [3.7, 4.1] | 20 | 3.9 [3.8, 4.1] |  |  |  |  |  |  |
|  | 48 h post-op | Mean (SD) | 60 | 3.8 (0.3) | 19 | 3.8 (0.3) | 21 | 3.7 (0.3) | 20 | 3.8 (0.4) |  |  |  |  |  |  |
|  |  | Median [IQR] | 60 | 3.8 [3.6, 4.0] | 19 | 3.9 [3.6, 4.0] | 21 | 3.7 [3.5, 3.9] | 20 | 3.8 [3.6, 4.0] |  |  |  |  |  |  |
| RAGE | pre-op | Mean (SD) | 60 | 3.4 (0.2) | 19 | 3.5 (0.1) | 21 | 3.4 (0.2) | 20 | 3.4 (0.2) | **<0.001** | 0.863 | 0.289 | 0.346 | 0.657 | 0.215 |
|  |  | Median [IQR] | 60 | 3.4 [3.3, 3.5] | 19 | 3.5 [3.4, 3.6] | 21 | 3.4 [3.3, 3.5] | 20 | 3.4 [3.2, 3.5] |  |  |  |  |  |  |
|  | 6 h post-op | Mean (SD) | 60 | 3.5 (0.3) | 19 | 3.5 (0.2) | 21 | 3.5 (0.3) | 20 | 3.5 (0.3) |  |  |  |  |  |  |
|  |  | Median [IQR] | 60 | 3.5 [3.3, 3.7] | 19 | 3.5 [3.4, 3.6] | 21 | 3.5 [3.3, 3.7] | 20 | 3.4 [3.3, 3.7] |  |  |  |  |  |  |
|  | 24 h post-op | Mean (SD) | 60 | 3.3 (0.3) | 19 | 3.4 (0.2) | 21 | 3.4 (0.3) | 20 | 3.3 (0.3) |  |  |  |  |  |  |
|  |  | Median [IQR] | 60 | 3.3 [3.2, 3.5] | 19 | 3.3 [3.2, 3.6] | 21 | 3.3 [3.2, 3.4] | 20 | 3.3 [3.1, 3.4] |  |  |  |  |  |  |
|  | 48 h post-op | Mean (SD) | 60 | 3.3 (0.3) | 19 | 3.3 (0.2) | 21 | 3.3 (0.3) | 20 | 3.3 (0.3) |  |  |  |  |  |  |
|  |  | Median [IQR] | 60 | 3.3 [3.1, 3.4] | 19 | 3.3 [3.1, 3.4] | 21 | 3.3 [3.1, 3.3] | 20 | 3.3 [3.0, 3.4] |  |  |  |  |  |  |
| SP-D | pre-op | Mean (SD) | 59 | 4.0 (0.2) | 18 | 4.2 (0.2) | 21 | 4.0 (0.2) | 20 | 3.9 (0.3) | **<0.001** | **0.0123** | 0.646 | 0.353 | 0.77 | 0.546 |
|  |  | Median [IQR] | 59 | 4.1 [3.9, 4.2] | 18 | 4.2 [4.0, 4.3] | 21 | 4.1 [3.9, 4.2] | 20 | 4.0 [3.7, 4.1] |  |  |  |  |  |  |
|  | 6 h post-op | Mean (SD) | 60 | 3.9 (0.2) | 19 | 4.0 (0.2) | 21 | 4.0 (0.2) | 20 | 3.8 (0.3) |  |  |  |  |  |  |
|  |  | Median [IQR] | 60 | 3.9 [3.8, 4.1] | 19 | 4.0 [3.8, 4.1] | 21 | 3.9 [3.8, 4.1] | 20 | 3.8 [3.6, 4.0] |  |  |  |  |  |  |
|  | 24 h post-op | Mean (SD) | 60 | 3.8 (0.2) | 19 | 3.9 (0.2) | 21 | 3.8 (0.2) | 20 | 3.7 (0.3) |  |  |  |  |  |  |
|  |  | Median [IQR] | 60 | 3.8 [3.7, 4.0] | 19 | 3.9 [3.8, 4.0] | 21 | 3.8 [3.7, 3.9] | 20 | 3.7 [3.5, 3.9] |  |  |  |  |  |  |
|  | 48 h post-op | Mean (SD) | 60 | 3.8 (0.2) | 19 | 3.9 (0.2) | 21 | 3.9 (0.2) | 20 | 3.7 (0.3) |  |  |  |  |  |  |
|  |  | Median [IQR] | 60 | 3.8 [3.7, 4.0] | 19 | 3.9 [3.7, 4.0] | 21 | 3.8 [3.8, 3.9] | 20 | 3.7 [3.6, 3.9] |  |  |  |  |  |  |
| Ang-1 | pre-op | Mean (SD) | 58 | 3.8 (0.3) | 18 | 3.8 (0.4) | 21 | 3.8 (0.2) | 19 | 3.8 (0.3) | **0.00965** | 0.0628 | 0.16 | 0.173 | 0.276 | 0.134 |
|  |  | Median [IQR] | 58 | 3.8 [3.6, 4.0] | 18 | 3.9 [3.6, 4.1] | 21 | 3.9 [3.6, 4.1] | 19 | 3.8 [3.5, 3.9] |  |  |  |  |  |  |
|  | 6 h post-op | Mean (SD) | 57 | 3.7 (0.3) | 17 | 3.7 (0.3) | 21 | 3.7 (0.3) | 19 | 3.7 (0.3) |  |  |  |  |  |  |
|  |  | Median [IQR] | 57 | 3.7 [3.5, 3.8] | 17 | 3.7 [3.5, 3.9] | 21 | 3.7 [3.5, 3.8] | 19 | 3.7 [3.5, 3.8] |  |  |  |  |  |  |
|  | 24 h post-op | Mean (SD) | 56 | 3.5 (0.3) | 16 | 3.6 (0.3) | 21 | 3.5 (0.4) | 19 | 3.5 (0.3) |  |  |  |  |  |  |
|  |  | Median [IQR] | 56 | 3.5 [3.3, 3.8] | 16 | 3.6 [3.5, 3.7] | 21 | 3.5 [3.3, 3.8] | 19 | 3.5 [3.3, 3.7] |  |  |  |  |  |  |
|  | 48 h post-op | Mean (SD) | 58 | 3.6 (0.3) | 18 | 3.7 (0.3) | 21 | 3.6 (0.4) | 19 | 3.4 (0.3) |  |  |  |  |  |  |
|  |  | Median [IQR] | 58 | 3.6 [3.3, 3.8] | 18 | 3.7 [3.6, 3.9] | 21 | 3.7 [3.4, 3.8] | 19 | 3.4 [3.2, 3.7] |  |  |  |  |  |  |
| Ang-2/Ang-1 | pre-op | Mean (SD) | 58 | 1.2 (1.5) | 18 | 1.2 (1.3) | 21 | 0.8 (0.9) | 19 | 1.5 (2.1) | **<0.001** | 0.223 | 0.654 | 0.364 | 0.442 | **<0.001** |
|  |  | Median [IQR] | 58 | 0.6 [0.4, 1.2] | 18 | 0.7 [0.5, 1.6] | 21 | 0.5 [0.3, 0.9] | 19 | 0.6 [0.4, 1.0] |  |  |  |  |  |  |
|  | 6 h post-op | Mean (SD) | 58 | 2.6 (3.2) | 18 | 2.7 (3.2) | 21 | 2.1 (2.2) | 19 | 3.1 (4.1) |  |  |  |  |  |  |
|  |  | Median [IQR] | 58 | 1.7 [0.9, 2.8] | 18 | 1.4 [0.9, 3.1] | 21 | 1.7 [1.0, 2.3] | 19 | 1.9 [1.0, 3.5] |  |  |  |  |  |  |
|  | 24 h post-op | Mean (SD) | 58 | 4.4 (5.9) | 18 | 3.2 (2.8) | 21 | 5.0 (8.0) | 19 | 5.0 (5.4) |  |  |  |  |  |  |
|  |  | Median [IQR] | 58 | 2.3 [1.3, 4.4] | 18 | 2.2 [1.5, 3.8] | 21 | 1.8 [1.1, 3.3] | 19 | 3.3 [1.5, 4.7] |  |  |  |  |  |  |
|  | 48 h post-op | Mean (SD) | 58 | 3.3 (4.2) | 18 | 2.4 (2.1) | 21 | 2.9 (4.9) | 19 | 4.5 (4.7) |  |  |  |  |  |  |
|  |  | Median [IQR] | 58 | 1.8 [0.6, 4.4] | 18 | 2.0 [0.6, 4.2] | 21 | 1.1 [0.7, 2.6] | 19 | 3.4 [0.9, 6.4] |  |  |  |  |  |  |

**Supplementary Table S6: Detailed results of blood sample analysis**

|  | **IL-6** | | | **Ang-2** | | |
| --- | --- | --- | --- | --- | --- | --- |
| *Predictors* | *Estimates* | *CI* | *p* | *Estimates* | *CI* | *p* |
| Intercept | 1.362 | 0.989 – 1.736 | **<0.001** | 1.162 | 0.500 – 1.825 | **0.001** |
| Baseline value | 0.295 | -0.085 – 0.675 | 0.127 | 0.751 | 0.577 – 0.926 | **<0.001** |
| Age [months] | -0.001 | -0.005 – 0.004 | 0.794 | -0.001 | -0.003 – 0.002 | 0.639 |
| Time [24 h post-op] | 0.021 | -0.321 – 0.364 | 0.902 | -0.208 | -0.761 – 0.345 | 0.458 |
| Time [48 h post-op] | -0.623 | -0.970 – -0.275 | **0.001** | -0.694 | -1.248 – -0.141 | **0.014** |
| Group [CPAP 5] | -0.095 | -0.324 – 0.134 | 0.413 | -0.018 | -0.129 – 0.094 | 0.756 |
| Group [LPV] | -0.065 | -0.293 – 0.162 | 0.570 | 0.019 | -0.087 – 0.125 | 0.718 |
| Group x Time [CPAP 5 x 24 h post-op] | 0.004 | -0.206 – 0.214 | 0.970 | -0.026 | -0.128 – 0.075 | 0.611 |
| Group x Time [CPAP 5 x 48 h post-op] | 0.043 | -0.170 – 0.255 | 0.693 | -0.031 | -0.133 – 0.072 | 0.554 |
| Group x Time [LPV x 24 h post-op] | -0.165 | -0.371 – 0.041 | 0.116 | -0.029 | -0.125 – 0.067 | 0.549 |
| Group x Time [LPV x 48 h post-op] | -0.015 | -0.224 – 0.195 | 0.890 | -0.038 | -0.135 – 0.060 | 0.444 |
| Baseline x Time [Baseline x 24 h post-op] | -0.111 | -0.455 – 0.233 | 0.525 | 0.069 | -0.079 – 0.218 | 0.358 |
| Baseline x Time [Baseline x 48 h post-op] | 0.241 | -0.108 – 0.589 | 0.174 | 0.170 | 0.021 – 0.319 | **0.025** |
| **Random Effects** | | | | | | |
| σ^2^ | 0.05 | | | 0.01 | | |
| τ_00_ | 0.07 _id_ | | | 0.02 _id_ | | |
| Observations | 160 | | | 160 | | |
| Marginal R^2^ / Conditional R^2^ | 0.272 / 0.693 | | | 0.692 / 0.874 | | |

|  | **RAGE** | | | **SP-D** | | |
| --- | --- | --- | --- | --- | --- | --- |
| *Predictors* | *Estimates* | *CI* | *p* | *Estimates* | *CI* | *p* |
| Intercept | 0.500 | -0.780 – 1.779 | 0.441 | 0.457 | -0.431 – 1.346 | 0.311 |
| Baseline value | 0.851 | 0.491 – 1.211 | **<0.001** | 0.835 | 0.625 – 1.045 | **<0.001** |
| Age [months] | -0.000 | -0.003 – 0.003 | 0.863 | 0.003 | 0.001 – 0.005 | **0.010** |
| Time [24 h post-op] | -0.335 | -1.286 – 0.616 | 0.488 | 0.079 | -0.734 – 0.892 | 0.849 |
| Time [48 h post-op] | 0.391 | -0.575 – 1.356 | 0.425 | 0.339 | -0.491 – 1.169 | 0.421 |
| Group [CPAP 5] | 0.140 | -0.024 – 0.305 | 0.094 | 0.065 | -0.051 – 0.182 | 0.269 |
| Group [LPV] | 0.114 | -0.045 – 0.273 | 0.159 | -0.014 | -0.135 – 0.106 | 0.813 |
| Group x Time [CPAP 5 x 24 h post-op] | -0.061 | -0.187 – 0.065 | 0.342 | -0.042 | -0.151 – 0.067 | 0.446 |
| Group x Time [CPAP 5 x 48 h post-op] | -0.031 | -0.159 – 0.096 | 0.628 | 0.024 | -0.086 – 0.135 | 0.663 |
| Group x Time [LPV x 24 h post-op] | -0.095 | -0.217 – 0.026 | 0.123 | 0.002 | -0.111 – 0.115 | 0.971 |
| Group x Time [LPV x 48 h post-op] | -0.042 | -0.166 – 0.081 | 0.499 | 0.037 | -0.077 – 0.151 | 0.524 |
| Baseline x Time [Baseline x 24 h post-op] | 0.074 | -0.196 – 0.344 | 0.589 | -0.041 | -0.235 – 0.153 | 0.674 |
| Baseline x Time [Baseline x 48 h post-op] | -0.166 | -0.440 – 0.108 | 0.233 | -0.110 | -0.307 – 0.088 | 0.275 |
| **Random Effects** | | | | | | |
| σ^2^ | 0.02 | | | 0.01 | | |
| τ_00_ | 0.04 _id_ | | | 0.02 _id_ | | |
| Observations | 160 | | | 160 | | |
| Marginal R^2^ / Conditional R^2^ | 0.353 / 0.811 | | | 0.566 / 0.809 | | |

|  | **Ang-1** | | | **Ang-2/Ang-1** | | |
| --- | --- | --- | --- | --- | --- | --- |
| *Predictors* | *Estimates* | *CI* | *p* | *Estimates* | *CI* | *p* |
| Intercept | 2.206 | 1.170 – 3.241 | **<0.001** | 1.662 | -0.415 – 3.739 | 0.116 |
| Baseline value | 0.379 | 0.112 – 0.646 | **0.006** | 1.068 | 0.431 – 1.704 | **0.001** |
| Age [months] | 0.003 | -0.000 – 0.007 | 0.059 | -0.027 | -0.069 – 0.016 | 0.219 |
| Time [24 h post-op] | -0.099 | -1.059 – 0.861 | 0.839 | -0.764 | -2.674 – 1.147 | 0.431 |
| Time [48 h post-op] | 0.791 | -0.181 – 1.763 | 0.110 | -0.080 | -2.019 – 1.860 | 0.936 |
| Group [CPAP 5] | -0.098 | -0.302 – 0.106 | 0.345 | 0.298 | -2.195 – 2.791 | 0.814 |
| Group [LPV] | -0.065 | -0.263 – 0.133 | 0.517 | 0.546 | -1.859 – 2.951 | 0.654 |
| Group x Time [CPAP 5 x 24 h post-op] | -0.024 | -0.213 – 0.166 | 0.805 | 1.957 | -0.531 – 4.445 | 0.122 |
| Group x Time [CPAP 5 x 48 h post-op] | 0.004 | -0.187 – 0.196 | 0.964 | 0.604 | -1.908 – 3.117 | 0.635 |
| Group x Time [LPV x 24 h post-op] | -0.108 | -0.291 – 0.074 | 0.244 | 1.047 | -1.337 – 3.432 | 0.387 |
| Group x Time [LPV x 48 h post-op] | -0.178 | -0.364 – 0.008 | 0.061 | 1.356 | -1.075 – 3.787 | 0.272 |
| Baseline x Time [Baseline x 24 h post-op] | 0.006 | -0.242 – 0.254 | 0.962 | 1.091 | 0.457 – 1.725 | **0.001** |
| Baseline x Time [Baseline x 48 h post-op] | -0.220 | -0.471 – 0.030 | 0.085 | 0.037 | -0.597 – 0.671 | 0.908 |
| **Random Effects** | | | | | | |
| σ^2^ | 0.04 | | | 6.66 | | |
| τ_00_ | 0.05 _id_ | | | 6.70 _id_ | | |
| Observations | 160 | | | 160 | | |
| Marginal R^2^ / Conditional R^2^ | 0.199 / 0.654 | | | 0.347 / 0.674 | | |

**Supplementary Table S7: Arterial blood gas analysis** performed preoperatively (pre-op) and postoperatively (post-op) across three intervention groups. Measured parameters include pH (mol L^-1^), partial pressure of carbon dioxide (paCO_2_; mmHg), partial pressure of oxygen (paO_2_; mmHg), arterial oxygen saturation (SaO_2_; %), base excess (BE; mmol L^-1^) and lactate (mmol L^-1^). The last column displays p-values from global F-tests in linear mixed-effects models, with values less than 0.05 shown in bold. Abbreviations: CPAP 5 = continuous positive airway pressure at 5 mbar, IQR = interquartile range, LPV = lung-protective ventilation, SD = standard deviation. Time*Group indicates the interaction between time and group. Absolute frequencies are denoted by n.

|  | | | **Total** | | **Apnea** | | **CPAP 5** | | **LPV** | | **p-values F-tests** | | | |
| --- | --- | --- | --- | --- | --- | --- | --- | --- | --- | --- | --- | --- | --- | --- |
| **Parameter** | **Time** | **Measure** | **n** |  | **n** |  | **n** |  | **n** |  | **Age** | **Time** | **Group** | **Time* Group** |
| pH (mol L^-1^) | pre-op | Mean (SD) | 60 | 7.4 (0.0) | 19 | 7.3 (0.0) | 21 | 7.3 (0.1) | 20 | 7.4 (0.0) | 0.528 | 0.223 | 0.848 | 0.145 |
|  |  | Median [IQR] | 60 | 7.4 [7.3, 7.4] | 19 | 7.4 [7.3, 7.4] | 21 | 7.3 [7.3, 7.4] | 20 | 7.4 [7.3, 7.4] |  |  |  |  |
|  | post-op | Mean (SD) | 60 | 7.3 (0.1) | 19 | 7.4 (0.1) | 21 | 7.3 (0.1) | 20 | 7.3 (0.1) |  |  |  |  |
|  |  | Median [IQR] | 60 | 7.3 [7.3, 7.4] | 19 | 7.3 [7.3, 7.4] | 21 | 7.3 [7.3, 7.4] | 20 | 7.3 [7.3, 7.4] |  |  |  |  |
| paCO_2_ (mmHg) | pre-op | Mean (SD) | 59 | 39.6 (5.4) | 19 | 40.4 (5.5) | 20 | 40.6 (5.8) | 20 | 37.8 (4.7) | 0.213 | **<0.001** | 0.617 | 0.0597 |
|  |  | Median [IQR] | 59 | 39.3 [35.8, 42.6] | 19 | 39.1 [36.0, 43.6] | 20 | 41.0 [37.4, 42.6] | 20 | 38.4 [34.6, 41.3] |  |  |  |  |
|  | post-op | Mean (SD) | 60 | 43.7 (6.4) | 19 | 41.6 (6.3) | 21 | 43.7 (6.8) | 20 | 45.7 (5.6) |  |  |  |  |
|  |  | Median [IQR] | 60 | 44.2 [39.9, 47.6] | 19 | 42.3 [37.6, 45.5] | 21 | 45.3 [38.8, 47.0] | 20 | 47.2 [42.0, 49.3] |  |  |  |  |
| paO_2_ (mmHg) | pre-op | Mean (SD) | 60 | 155.7 (100.0) | 19 | 139.6 (87.0) | 21 | 165.1 (129.3) | 20 | 161.0 (76.8) | 0.296 | 0.17 | 0.716 | 0.454 |
|  |  | Median [IQR] | 60 | 127.6 [94.5, 176.8] | 19 | 113.3 [84.6, 158.9] | 21 | 115.4 [66.8, 201.9] | 20 | 135.7 [111.8, 170.9] |  |  |  |  |
|  | post-op | Mean (SD) | 60 | 135.0 (86.1) | 19 | 142.3 (112.3) | 21 | 139.4 (70.7) | 20 | 123.6 (74.8) |  |  |  |  |
|  |  | Median [IQR] | 60 | 102.7 [78.2, 156.9] | 19 | 102.5 [74.7, 151.5] | 21 | 114.3 [88.9, 156.8] | 20 | 99.2 [87.7, 137.1] |  |  |  |  |
| SaO_2_ (%) | pre-op | Mean (SD) | 60 | 96.4 (4.5) | 19 | 95.7 (5.4) | 21 | 95.3 (5.3) | 20 | 98.4 (0.9) | 0.981 | 0.45 | 0.393 | 0.215 |
|  |  | Median [IQR] | 60 | 98.2 [96.4, 99.0] | 19 | 97.8 [95.8, 99.1] | 21 | 97.8 [93.0, 98.9] | 20 | 98.7 [97.9, 99.0] |  |  |  |  |
|  | post-op | Mean (SD) | 60 | 96.3 (4.2) | 19 | 95.3 (6.2) | 21 | 96.9 (2.9) | 20 | 96.5 (2.5) |  |  |  |  |
|  |  | Median [IQR] | 60 | 97.7 [95.2, 98.7] | 19 | 97.7 [95.0, 98.6] | 21 | 98.0 [94.8, 98.9] | 20 | 97.0 [95.8, 98.4] |  |  |  |  |
| BE (mmol L^-1^) | pre-op | Mean (SD) | 59 | -3.8 (2.1) | 19 | -3.8 (2.2) | 20 | -4.4 (2.2) | 20 | -3.2 (1.6) | 0.872 | **0.0259** | 0.539 | 0.907 |
|  |  | Median [IQR] | 59 | -3.6 [-5.0, -2.2] | 19 | -3.6 [-5.2, -2.0] | 20 | -4.4 [-6.2, -2.6] | 20 | -3.3 [-4.4, -2.1] |  |  |  |  |
|  | post-op | Mean (SD) | 60 | -2.6 (2.6) | 19 | -2.9 (3.0) | 21 | -2.5 (2.6) | 20 | -2.4 (2.4) |  |  |  |  |
|  |  | Median [IQR] | 60 | -2.9 [-3.7, -1.1] | 19 | -3.2 [-4.2, -2.0] | 21 | -2.9 [-3.5, -0.4] | 20 | -2.5 [-3.4, -1.0] |  |  |  |  |
| Lactate (mmol L^-1^) | pre-op | Mean (SD) | 60 | 1.0 (0.3) | 19 | 1.0 (0.4) | 21 | 0.9 (0.2) | 20 | 1.0 (0.3) | **0.00469** | **<0.001** | 0.993 | 0.989 |
|  |  | Median [IQR] | 60 | 0.9 [0.8, 1.1] | 19 | 0.9 [0.8, 1.1] | 21 | 0.9 [0.8, 1.0] | 20 | 0.9 [0.8, 1.1] |  |  |  |  |
|  | post-op | Mean (SD) | 58 | 2.1 (1.1) | 17 | 2.2 (1.2) | 21 | 1.9 (1.0) | 20 | 2.1 (1.1) |  |  |  |  |
|  |  | Median [IQR] | 58 | 1.7 [1.4, 2.5] | 17 | 2.0 [1.6, 2.5] | 21 | 1.5 [1.2, 2.5] | 20 | 2.0 [1.5, 2.3] |  |  |  |  |

**Supplementary Table S8: Arterial blood gas analysis – statistical results**

|  | **pH (mol L^-1^)** | | | **paCO_2_ (mmHg)** | | |
| --- | --- | --- | --- | --- | --- | --- |
| *Predictors* | *Estimates* | *CI* | *p* | *Estimates* | *CI* | *p* |
| Intercept | 7.349 | 7.320 – 7.377 | **<0.001** | 39.580 | 36.551 – 42.609 | **<0.001** |
| Age [months] | -0.000 | -0.001 – 0.000 | 0.528 | 0.038 | -0.022 – 0.097 | 0.213 |
| Time [post-op] | 0.004 | -0.033 – 0.042 | 0.820 | 1.882 | -2.133 – 5.898 | 0.355 |
| Group [CPAP 5] | 0.000 | -0.037 – 0.038 | 0.983 | 0.960 | -3.119 – 5.039 | 0.642 |
| Group [LPV] | 0.028 | -0.008 – 0.064 | 0.131 | -2.668 | -6.549 – 1.212 | 0.176 |
| Time x Group [post-op x CPAP 5] | -0.006 | -0.059 – 0.047 | 0.828 | 0.809 | -4.914 – 6.532 | 0.780 |
| Time x Group [post-op x LPV] | -0.046 | -0.097 – 0.005 | 0.075 | 6.038 | 0.576 – 11.499 | **0.031** |
| **Random Effects** | | | | | | |
| σ^2^ | 0.00 | | | 34.80 | | |
| τ_00_ | 0.00 _id_ | | | 0.00 _id_ | | |
| Observations | 108 | | | 107 | | |
| Marginal R^2^ / Conditional R^2^ | 0.057 / NA | | | 0.180 / NA | | |

|  | **paO_2_ (mmHg)** | | | **SaO2 (%)** | | |
| --- | --- | --- | --- | --- | --- | --- |
| *Predictors* | *Estimates* | *CI* | *p* | *Estimates* | *CI* | *p* |
| Intercept | 135.064 | 85.225 – 184.902 | **<0.001** | 96.244 | 94.358 – 98.130 | **<0.001** |
| Age [months] | 0.542 | -0.476 – 1.559 | 0.293 | 0.000 | -0.041 – 0.042 | 0.981 |
| Time [post-op] | 7.447 | -55.349 – 70.243 | 0.814 | 0.135 | -1.902 – 2.173 | 0.895 |
| Group [CPAP 5] | 35.642 | -30.119 – 101.402 | 0.285 | -0.166 | -2.622 – 2.290 | 0.894 |
| Group [LPV] | 12.848 | -50.695 – 76.391 | 0.689 | 2.119 | -0.257 – 4.495 | 0.080 |
| Time x Group [post-op x CPAP 5] | -51.765 | -140.572 – 37.043 | 0.250 | 0.259 | -2.623 – 3.141 | 0.859 |
| Time x Group [post-op x LPV] | -44.857 | -130.269 – 40.555 | 0.300 | -1.985 | -4.757 – 0.786 | 0.158 |
| **Random Effects** | | | | | | |
| σ^2^ | 8513.49 | | | 8.96 | | |
| τ_00_ | 811.58 _id_ | | | 4.04 _id_ | | |
| Observations | 108 | | | 108 | | |
| Marginal R^2^ / Conditional R^2^ | 0.046 / 0.129 | | | 0.047 / 0.343 | | |

|  | **BE (mmol L^-1^)** | | | **Lactate (mmol L^-1^)** | | |
| --- | --- | --- | --- | --- | --- | --- |
| *Predictors* | *Estimates* | *CI* | *p* | *Estimates* | *CI* | *p* |
| Intercept | -3.953 | -5.178 – -2.728 | **<0.001** | 0.756 | 0.383 – 1.128 | **<0.001** |
| Age [months] | 0.002 | -0.022 – 0.026 | 0.872 | 0.012 | 0.004 – 0.019 | **0.004** |
| Time [post-op] | 1.000 | -0.612 – 2.612 | 0.221 | 1.085 | 0.611 – 1.560 | **<0.001** |
| Group [CPAP 5] | 0.016 | -1.633 – 1.664 | 0.985 | 0.005 | -0.485 – 0.495 | 0.984 |
| Group [LPV] | 0.690 | -0.878 – 2.258 | 0.385 | -0.035 | -0.509 – 0.438 | 0.882 |
| Time x Group [post-op x CPAP 5] | 0.327 | -1.970 – 2.624 | 0.778 | 0.005 | -0.655 – 0.664 | 0.989 |
| Time x Group [post-op x LPV] | -0.160 | -2.352 – 2.032 | 0.885 | 0.043 | -0.592 – 0.677 | 0.894 |
| **Random Effects** | | | | | | |
| σ^2^ | 5.61 | | | 0.45 | | |
| τ_00_ | 0.08 _id_ | | | 0.07 _id_ | | |
| Observations | 107 | | | 106 | | |
| Marginal R^2^ / Conditional R^2^ | 0.059 / 0.072 | | | 0.410 / 0.485 | | |

**Supplementary Figure S1:** **CONSORT flowchart**. Abbreviations: CPAP 5 = continuous positive airway pressure at 5 mbar, ICU = intensive care unit, LPV = lung-protective ventilation. Absolute frequencies are denoted by n.

**LIMITATIONS – Sample size**

To inform the design of future studies, we performed a sample size calculation using the difference between pre-operative and post-operative spontaneous breathing CoVy values as the outcome in an one-way ANOVA with group as the independent variable. Given the limited size of the pilot sample, we employed a bootstrap approach to estimate the effect size (Cohen’s f) and its 95% confidence interval, yielding a mean effect size of 0.23 (medium effect) with a wide confidence interval ranging from 0.04 (small effect) to 0.50 (large effect). Based on these estimates, the required sample size to achieve 80% power at a 5% significance level ranges from 14 to 2009 patients per group (42 to 6027 in total), reflecting the uncertainty inherent in pilot data. While this approach provides a transparent and data-driven starting point for sample size planning, the wide confidence intervals highlight the imprecision of this pilot estimate. Therefore, these results should be interpreted with caution and complemented by clinical judgment and evidence from previous studies when finalizing sample size requirements.
